# 740 Injury Prevention Program Targeting Tap Water Scald Burns in Pediatric Patients

**DOI:** 10.1093/jbcr/irae036.283

**Published:** 2024-04-17

**Authors:** Lisa C Vitale, Renee Zarr, Mariah Malaniak

**Affiliations:** Children's Hospital of Michigan, Detroit, MI; Children's Hospital of Michigan, Detroit, MI; Children's Hospital of Michigan, Detroit, MI

## Abstract

**Introduction:**

Children in our urban ABA verified pediatric burn center have low socioeconomic status: 57.6% live in poverty, 67.9% receive food assistance and 65% are Medicaid-eligible. Research has consistently shown a correlation between childhood poverty and increased risk of unintentional injury and death. Using demographic and injury incidence data, our Injury Prevention Program developed education on scald burn prevention targeting expectant mothers and families with children ≤ 5 years.

**Methods:**

A safety assessment was completed by caregivers and responses evaluated to customize the safety education. A tailored safety educational workshop was created based on burn registry data and pre assessment responses from caregivers. An immediate post workshop follow-up assessment was given to the caregivers about their educational workshop gauging their knowledge retention and comprehension. A one-month post assessment is then given to at least 25% of workshop attendees to assess knowledge retention.

**Results:**

Caregiver pre and post comprehension assessments were evaluated to determine the program’s overall effectiveness. In 2022, the program pre-test assessment scores averaged 40%. Post-test assessment scores then improved to an average of 84%. P-Values were also calculated to determine that the injury prevention program was highly statistically significant with all correct assessment responses having a P-Value of < .0001. At one-month post course, caregivers are re-assessed for knowledge retention. Of those re-evaluated 94% of participants had retained critical knowledge and were noted to score ≥80% on one month post course evaluation.

**Conclusions:**

It is well known that scald burns are the most common pediatric burn injury. From 2021-2022 there was a 10% decrease in patients presenting to our center with tap water scald injuries. This data suggests a targeted injury prevention program may help to increase caregiver knowledge and decrease common pediatric burn injuries.

**Applicability of Research to Practice:**

Regular evaluation of prevention programs is critical in assessing for effectiveness. More research is needed to understand parental behavior pre and post course, along with knowledge retention to maximize efficiency of injury prevention programs.

